# Neutralizing antidrug antibody to emicizumab in patients with severe hemophilia A: Case report of a first noninhibitor patient and review of the literature

**DOI:** 10.1016/j.rpth.2023.102194

**Published:** 2023-08-30

**Authors:** Hande Kizilocak, Michael F. Guerrera, Guy Young

**Affiliations:** 1 Children’s Hospital Los Angeles, Hemostasis and Thrombosis Center, Los Angeles, CA, USA; 2 Children’s National Hospital, Washington, DC, USA; 3 University of Southern California, Keck School of Medicine, Los Angeles, CA, USA

**Keywords:** antidrug antibodies, case report, emicizumab, factor VIII, hemophilia

## Abstract

**Background:**

Hemophilia A (HA) is a genetic bleeding disorder characterized by the deficiency of the coagulation protein factor (F) VIII (FVIII). The development of neutralizing antidrug antibodies (ADAs) to factor concentrates (inhibitors) created an unmet need for novel therapies. The first agent to address this need is emicizumab.

**Key Clinical Question:**

Can emicizumab ADA occur in patients with HA without FVIII inhibitors?

**Clinical Approach:**

A new case (the first in a noninhibitor patient) presented with unexpected and excessive bleeding and a prolonged activated partial thromboplastin time. The patient was evaluated by assessing FVIII levels, and the previously published modified version of the Bethesda assay was used to determine the level of ADA to emicizumab.

**Conclusion:**

Although emicizumab is very effective and has minimal immunogenicity, ADAs, albeit rare, can still occur. There have been 4 previously published anti-emicizumab ADA cases with severe HA with inhibitors, and herein, we describe 1 new case with severe HA without inhibitors.

## Essentials

• Patients receiving emicizumab can develop antibodies that block emicizumab from working.
• The first sign of an emicizumab antidrug antibody can be unexpected or excessive bleeding.
• There is a blood test that can be used to determine if a patient has antibodies to emicizumab.
• Health care workers should ask patients to undergo that blood test if an antibody is suspected.

## 1 Introduction

Hemophilia A (HA) is a genetic bleeding disorder characterized by the deficiency of factor (F) VIII (FVIII). For decades, the standard treatment has been factor replacement using clotting factor concentrates (CFCs). Aside from the catastrophic transmission of infectious diseases which led to the development of recombinant CFCs, the most serious adverse effect of CFCs is immunogenicity. This results in so-called inhibitors, antidrug antibodies (ADAs), against FVIII which significantly complicate patient management [1]. This serious issue was the main driver for the development of novel therapies aimed at addressing the major treatment gap between noninhibitor and inhibitor patients. The first such agent is emicizumab (Hemlibra, Roche), a humanized bispecific monoclonal antibody that mimics the procoagulant activity of FVIII by bridging activated FIX to FX [2]. It has been approved for prophylaxis in people with HA with and without inhibitors and has been shown to be safe and efficacious in the prevention of bleeding in the HAVEN clinical trials [[2], [3], [4], [5]].

Considering this history, a key safety measure for all new agents in hemophilia is immunogenicity. Fortunately, emicizumab ADAs are very rare. Nevertheless, understanding in whom and when these occur and how to identify these ADAs is an important aspect in the clinical management of patients receiving emicizumab. In the HAVEN clinical trials, 0.75% (3/398) of subjects developed ADAs with neutralizing potential [6] including 2 children in the HAVEN 2 pediatric trial [3]. As of February 2023, there are 4 reported cases with ADAs to emicizumab [[7], [8], [9], [10]]. Herein we report a new case of emicizumab ADA (including the first in a patient without FVIII inhibitors), review how to identify these ADAs and review the previously published cases.

## 2 Case Presentation and Diagnostic Assessment

As a result of our previous publication demonstrating a novel method for detecting emicizumab ADA using commercially available materials [10], we have been contacted by other clinicians to determine whether their patients have an anti-emicizumab ADA. During this process, we also collected relevant clinical information on these patients. For each new case, we requested freshly frozen citrated plasma which was received frozen on dry ice and stored at −80°C. Each sample was thawed, and we then performed an activated partial thromboplastin time (aPTT) and FVIII levels using a one-stage clotting assay (OSCA) with SynthASil on the ACL TOP 500 (Instrumentation Laboratory). FVIII activity was also determined photometrically via the Chromogenix Coatest SP4 FVIII chromogenic assay kit (bovine chromogenic FVIII activity [bovCHR], Diapharma Group) and the Biophen FVIII:C chromogenic assay kit (human chromogenic FVIII activity [humCHR] Aniara Diagnostica). For the modified Bethesda assay for emicizumab, see the prior publication for details [10]. Briefly, emicizumab control plasma was collected from patients in our clinic who were on stable doses of emicizumab. Once this was available, 200 μL of patient plasma containing the suspected ADA to emicizumab was added to a polypropylene test tube neat or diluted (1:2, 1:4, 1:8, 1:16, 1:32) with imidazole buffer (3.4 g/L imidazole, pH 7.5 ± 0.5; Siemens Healthcare Diagnostics’ Newark). To each tube, 200 μL of plasma with a known humCHR activity was added. A control tube was also prepared, containing 200 μL of FVIII-deficient plasma (Instrumentation Laboratory) and 200 μL of emicizumab-containing plasma. After incubation, each sample was tested for FVIII activity using the humCHR assay. The resulting values were then used to calculate the titer of the inhibitor expressed in Bethesda units. Lastly, we performed a detailed literature search using the following terms “emicizumab, antidrug antibody, ADA, and immunogenicity,” to identify other published cases which we have aggregated in the results section.

The new case is a 5-year-old male with severe HA without inhibitors with intron 22 inversion. Once weekly recombinant FVIII prophylaxis was commenced at 9 months of age and switched to twice weekly at 15 months. He remained on this prophylaxis regimen until 5 years of age when the prophylaxis switched to emicizumab. At the time of the switch, he had >150 exposure days and it was a shared decision with the family. Annual bleeding rate prior to the switch and after the switch were 2 and 0, respectively. Due to the clinical suspicion of a loss of response to emicizumab (left knee hemarthrosis, post traumatic left hamstring hematoma, and right ankle hemarthrosis at weeks 5, 8, and week 11 after the initial emicizumab bolus dose, respectively), an aPTT was performed at the patient’s local laboratory at week 11 and was found to be markedly prolonged. Following this finding, additional blood samples were sent to the Children’s Hospital Los Angeles special coagulation laboratory for a more detailed characterization of the presumed ADA. The patient had a prolonged aPTT, a bovCHR FVIII level <1%, and unexpectedly a decreased OSCA FVIII and humCHR FVIII activity levels (Table). In order to further assess the inhibitory effect of the patient’s anti-emicizumab antibody, the modified Bethesda assay was performed with a result of 1.1 Bethesda Units. Emicizumab prophylaxis was discontinued for this patient and FVIII prophylaxis was re-initiated. Of note, the patient has no history of infection, vaccination, or autoimmune disorders at the time of ADA development and has 2 brothers on emicizumab prophylaxis, one with a history of transient FVIII inhibitor.

**Table:** Laboratory findings of the new case and review of the literature.

Cases	Age (y)	Symptoms suggesting ADA (postemicizumab initiation)	Timing of ADA identification (postemicizumab initiation)	aPTT	OSCA FVIII level	BovCHR FVIII level	HumCHR FVIII level
Case-A	13	Week-5	Week-5	↑	↓	Not reported	↓
Case-B	62	Week-41	Week-49	↑	Not reported	Not reported	Not reported
Case-C	2	Week-24	Week-24	↑	Normal	Not reported	Normal
Case-D	7	Week-16	Week-20	↑	↓	↓	↓
New case	5	Week-5	Week-11	↑	↓	↓	↓

ADA, antidrug antibody; aPTT, activated partial thromboplastin time; BovCHR, bovine chromogenic; HumCHR, human chromogenic; OSCA, one-stage clotting assay.

The first reported case (A) in the literature was a 13-year-old male with severe HA with inhibitors who was a participant of the HAVEN 2 trial [7]. He experienced bleeding symptoms at week 5 of emicizumab therapy, which raised suspicion of a loss of response to emicizumab. Pharmacokinetic studies at week 5 revealed a prolonged aPTT and undetectable humCHR FVIII activity. Since he was a HAVEN 2 trial participant, the emicizumab level was measured and was undetectable. Despite doubling the dose of emicizumab, he had no improvement and was switched back to bypassing agent prophylaxis. The second reported case (B) was a 62-year-old male with severe HA with inhibitors who started to develop bleeding symptoms at week 41 of emicizumab prophylaxis which was then stopped at week 49 after detecting a prolongation of aPTT [8]. The third reported case (C) was a 2-year-old male with severe HA with inhibitors who had unexpected bleeding symptoms and a prolonged aPTT at week-24 of emicizumab prophylaxis [9]. However, unlike the other cases, his OSCA FVIII level and humCHR FVIII activity were normal. The reason for this difference is that this patient has a nonneutralizing ADA which increased emicizumab clearance, whereas the other cases had a neutralizing emicizumab ADA. Upon further evaluation, using immunosorbent assays, the authors demonstrated rapid emicizumab clearance when the antibody was tested in *in vivo* mice experiments. The fourth reported case (D) was a 7-year-old male with severe HA with inhibitors [10]. He first presented with an increase in bruising at week 16 of emicizumab and experienced a traumatic muscle hematoma of right upper extremity with the prolongation of aPTT at week 20 of emicizumab prophylaxis. This is the previously published case that led to the development of the modified Bethesda [10]. Despite doubling the emicizumab dose, his symptoms persisted, and the patient switched to daily bypassing agent prophylaxis.

## 3 Discussion

Emicizumab has been a revolutionary new medication in hemophilia and has become the most commonly prescribed prophylactic treatment [11]. Nevertheless, there have been cases of clinically important emicizumab ADA reported, and while these are very rare, it is incumbent upon clinicians to be aware not only that these can occur but importantly how to recognize them. The first key feature is that, thus far, all the reported cases including the new one described here presented with unexpected bleeding. Second, laboratory testing demonstrated a prolonged aPTT in all the cases. Third, all of these cases were identified in the first year following initiation of treatment and with the exception of the case from Japan, they were all identified within weeks of initiation of emicizumab. Finally, until this report, all the prior published cases were in patients with FVIII inhibitors suggesting that these patients’ immune systems are primed to make antibodies against therapeutic proteins. A key addition of this report is the demonstration that anti-emicizumab ADA can occur in noninhibitor patients.

It is important to emphasize that emicizumab is a very effective drug with minimal immunogenicity. With the new case in this report, the total number of known cases is 5 out an approximately 19,000 patients [12] who have received emicizumab worldwide, and thus ADAs to emicizumab are very rare. Importantly, this may be an underestimate as there are probably other cases that have neither not been reported nor come to our attention, but, nevertheless, given that such ADAs result in dramatic changes to a patient’s treatment, it is likely that whatever missed cases there may be are likely to be small. The most important lesson from these cases is first, that most of the emicizumab ADAs were reported in the first 25 weeks of emicizumab exposure (4 out of 5 cases). On the positive side, it strongly suggests that patients who have been receiving emicizumab for >6 months are very unlikely to develop an ADA; however, this also means that prescribers should ensure close follow-up after initiating emicizumab, especially in the first 6 months. Second, all cases initially presented with prolonged aPTT, a test that is readily available and can offer strong suggestion in the right clinical context that the patient has an ADA. Finally, a variety of assays were used in the reported cases to detect the ADAs raising the clear need for the development of either a commercially available validated assay (ideal) or at least a consistent, perhaps standardized, laboratory-developed test that special coagulation laboratories can perform.
